# Changes in the *Arabidopsis thaliana* Proteome Implicate cAMP in Biotic and Abiotic Stress Responses and Changes in Energy Metabolism

**DOI:** 10.3390/ijms17060852

**Published:** 2016-06-01

**Authors:** May Alqurashi, Chris Gehring, Claudius Marondedze

**Affiliations:** 1 Biological and Environmental Sciences and Engineering Division, King Abdullah University of Science and Technology, Thuwal 23955-6900, Saudi Arabia; may.qurashi@kaust.edu.sa (M.A.); christoph.gehring@kaust.edu.sa (C.G.); 2 Cambridge Centre for Proteomics, Cambridge System Biology, Department of Biochemistry, University of Cambridge, Tennis Court Road, Cambridge CB2 1QR, UK

**Keywords:** cAMP-dependent proteome, biotic stress, abiotic stress, glycolysis, TCA cycle

## Abstract

The second messenger 3′,5′-cyclic adenosine monophosphate (cAMP) is increasingly recognized as having many different roles in plant responses to environmental stimuli. To gain further insights into these roles, *Arabidopsis* *thaliana* cell suspension culture was treated with 100 nM of cell permeant 8-bromo-cAMP for 5 or 10 min. Here, applying mass spectrometry and comparative proteomics, 20 proteins were identified as differentially expressed and we noted a specific bias in proteins with a role in abiotic stress, particularly cold and salinity, biotic stress as well as proteins with a role in glycolysis. These findings suggest that cAMP is sufficient to elicit specific stress responses that may in turn induce complex changes to cellular energy homeostasis.

## 1. Introduction

Environmental factors such as biotic and abiotic stresses can cause constraints on the growth, development and productivity of plants. These stresses also disturb cellular homeostasis, and consequently, a rapid response is initiated to alleviate the impact of stress. Signaling molecules including second messengers such as cyclic nucleotides play an important role in this early phase. Cyclic nucleotides and 3′,5′-cyclic adenosine monophosphate (cAMP) in particular have long been established as important messengers in prokaryotes as well as in lower and higher eukaryotes [1]. In plants, cAMP has been reported to have direct and/or indirect roles in many developmental processes including pollen growth [2] and response to biotic stress [3,4,5].

**Figure 1:**
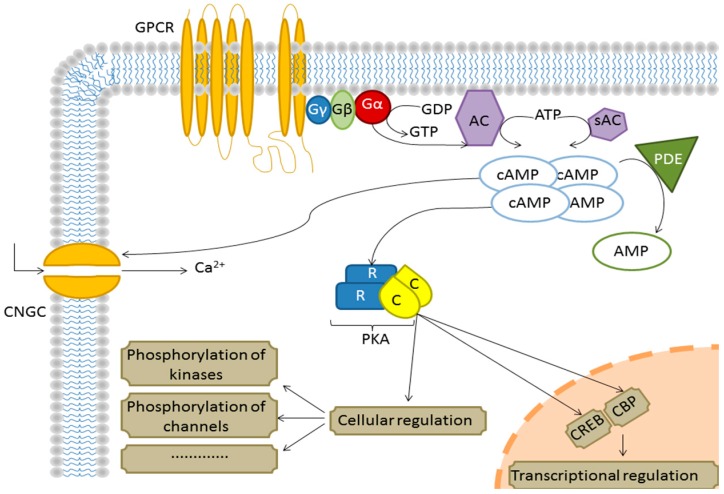
An illustration of some signal transduction pathways mediated by the G-protein coupled receptor based on the animal system. When adenylyl cyclase (AC) is activated by the Gα-subunit of the G-protein coupled receptor (GPCR), it catalyzes the formation of cAMP. Cyclic AMP then activates many substrates and kinases such as protein kinase A (PKA) which will regulate many biological processes. Cyclic nucleotide-gated channel (CNGC); soluble adenylyl cyclase (sAC); phosphodiesterase (PDE); cAMP response element-binding (CREB); CREB-binding protein (CBP); catalytic subunit of PKA (C); regulatory subunit of PKA (R).

The levels of cAMP have been shown to increase in response to biotic stress and subsequently influence calcium (Ca^2+^) influx by targeting membrane cyclic nucleotide-gated channels (CNGCs) [6], thereby increasing cytosolic free Ca^2+^. Plant CNGCs are ligand and voltage-gated channels functioning in sensory signal transduction and have been proposed to regulate Ca^2+^ influx into the cytosol [7,8]. The CNGCs also have a role in plant development and plant responses to biotic and abiotic stress [9]. CNGCs open upon binding of either cAMP or 3′,5′-cyclic guanosine monophosphate (cGMP) which act as potential activating ligands [8] and close upon binding of Ca^2+^/calmodulin (Figure 1). The cyclic nucleotides and calmodulin competitively bind to overlapping binding sites at the C-terminus in the cytosolic part of the channel [10,11]. In turn, Ca^2+^ amplifies the signal as part of the cellular response [12]. In *Arabidopsis*, there are 20 annotated CNGCs [13,14] and CNGC2 in particular is an inward-rectifying potassium (K^2+^) channel that is blocked by Ca^2+^ [8]. Mutation of CNGC2 results in an impaired hypersensitive response to avirulent pathogens and cAMP-/cGMP-dependent cytosolic Ca^2+^ elevation [15].

At the structural level, voltage-independent channels (VICs) in the plasma membrane of *Arabidopsis* root cells have been reported to be highly sensitive to cAMP and these channels are also implicated in salt tolerance in *Arabidopsis* seedlings by reducing sodium (Na^+^) influx [16]. Furthermore, an increasing number of plant mononucleotide cyclases, including adenylate cyclases (enzymes that synthesis cAMP from ATP), have been predicted [17,18] and experimentally confirmed [2,19].

Mass spectrometry-based approaches have been employed previously to study cAMP in plants, firstly to determine the concentration of cAMP [20], secondly to look at the interactome network of cAMP [21], and finally to look at the cAMP-dependent responses [22]. In the latter study, systems-level analyses showed that upon treatment of *Arabidopsis* plants with 1 or 10 µM of cAMP, proteins involved in responses to temperature, light and photosynthesis were changing in abundance at 1 and 3 h after treatment. However, previous studies have used high concentrations of cAMP, which may not be physiologically relevant. Therefore, we have undertaken studying cAMP-dependent proteome changes at low cAMP concentration and monitoring early cellular responses with a view to gain further insight into the possible physiological implications at cellular levels.

## 2. Results and Discussion

In order to obtain a cAMP-dependent proteome, Arabidopsis ecotype Columbia-0 (Col-0) cell suspension cultures were treated with 100 nM of cell permeant 8-Br-cAMP and samples were collected at 5 and 10 min after treatment. Extracted proteins from three biological replicates were digested with trypsin and the peptides were labeled with tandem mass tag (TMT) six-plex for quantitative analysis. A total of 1023 quantifiable proteins were identified (false discovery rate: 0.8%). Proteins present in at least two biological replicates from each treatment time point were compared to their corresponding controls. Twenty proteins were detected as differentially expressed, with at least a ±1.5-fold change (±0.6 in log_2_ transformation) and a statistically significant *p*-value of ≤0.05 (Table 1). After 5 min of cAMP treatment, three proteins increased in abundance and four proteins decreased in abundance, while after 10 min of cAMP treatment, 10 proteins increased in abundance and four proteins decreased in abundance. In this study, only a few of the proteins fulfilled the set thresholds and it is of particular note that in addition to each protein being detected in at least two out of three biological replicates, proteins from cAMP-treated cells were compared with mock-treated cells collected at the same time point.

**Figure 2:**
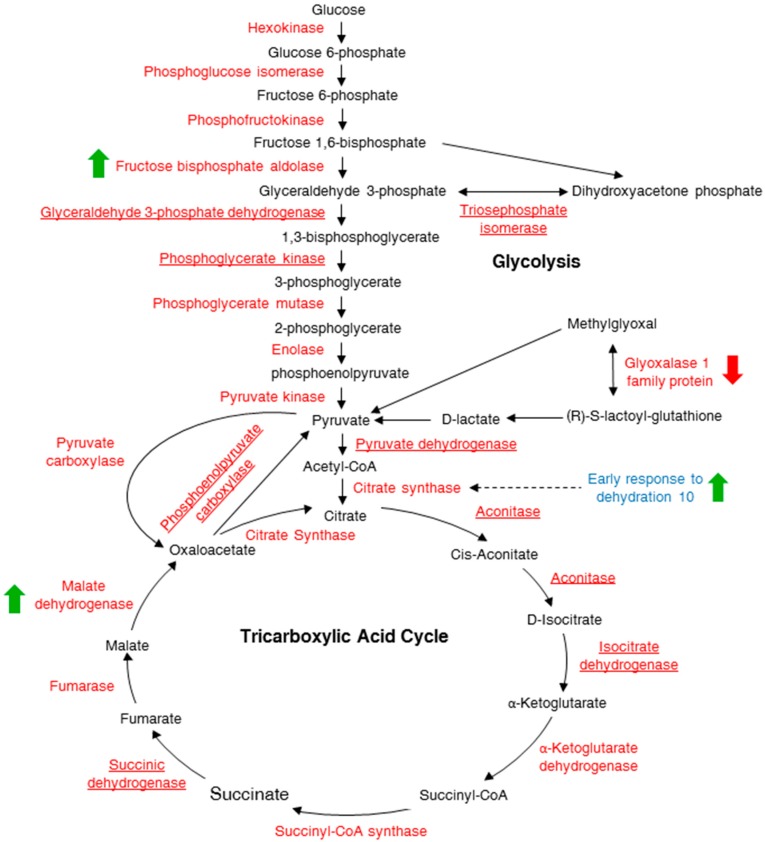
A schematic diagram of the glycolysis and tricarboxylic acid (TCA) cycle pathways showing proteins differentially expressed post cAMP treatment. The glycolysis and TCA metabolic products are shown in black, the enzymes are in red and a protein associated with the TCA cycle is shown in blue. The green arrows indicate proteins that accumulate in response to cAMP while the red arrow indicates a protein that is reduced in quantity. All the other proteins underlined are some of the proteins identified in the current study that are not changing in abundance after cAMP treatment.

Gene ontology (GO) analysis using FatiGO^+^ allowed classification of identified proteins in each group based on their enrichments as compared to the normal distribution [23,24]. Eight of the 20 differentially expressed proteins are in the category “response to cold”, six proteins are in the category “response to salt stress”, five proteins are in both “response to bacterium” and “response to cadmium ion” and four proteins are enriched in the category “glycolytic process” (Table 1). Although 24 proteins that are involved in the glycolytic process were identified (Table S1), only four were significantly changing in abundance at either 5 or 10 min after cAMP treatment. The four proteins include fructose-bisphosphate aldolase 3 (FBA3; At2g01140), mitochondrial malate dehydrogenase (MDH; At1g53240) and early response to dehydration 10 (ERD10; At1g20450) which increased in abundance while glyoxalase I (At1g08110) decreased (Figure 2).

Fructose-bisphosphate aldolase catalyzes two reversible reactions. The first involves the enzymatic conversion of fructose 1,6-bisphosphate into the triose phosphates, dihydroxyacetone phosphate and glyceraldehyde 3-phosphate in gluconeogenesis. The second reaction involves the condensation reaction of fructose-1,6-biphosphate and sedoheptulose-1,7-biphosphate in the Calvin cycle [25]. The mRNA levels of FBA3 have been shown to increase in response to abscisic acid (ABA) and salicylic acid (SA) and to various abiotic stresses such as salinity, drought, cold and heat, but the expression levels were noted to decrease in response to cadmium (Cd^2+^) ions [26]. Increased abundance of FBA3 was also observed at 5 min after cAMP treatment (Table 1) and this implicates cAMP in abiotic stress responses.

Malate dehydrogenase reversibly catalyzes the oxidation of malate to oxaloacetate using the reduction of NAD^+^ to NADH in the tricarboxylic acid (TCA) cycle [27]. The control of the TCA cycle activity is dependent on several enzymes, including aconitase, fumarase, succinate dehydrogenase, 2-oxoglutarate dehydrogenase and MDH, which all have different flux control coefficients [28]. A flux control coefficient is a quantifiable parameter measuring the contribution or effect of enzymes on the overall steady-state flux of a metabolic pathway. Mitochondrial MDH has the highest flux control coefficient (1.76) and is considered one of the rate-limiting steps [28]. Plant mitochondrial MDH is also important in oxidizing NADH in the TCA cycle and in the malate/aspartate shuttle in photorespiration [29]. Malate dehydrogenase has been shown to increase in abundance in response to oxidative stress [30] and flooding [31]. In tomato (*Solanum lycopersicum*), mutations in mitochondrial MDH affect growth and fruit yield [32], while in *Arabidopsis*, single and double knockouts of the two mitochondrial *MDH* genes appear to have little effect on growth and development [33]. In addition to MDH increasing in abundance in response to cAMP, ERD10 also increased in abundance in response to cAMP treatment (Table 1). An increase in abundance of ERD10 has previously been shown to have a protective role by slowing the heat-induced aggregation and/or inactivation rate of various substrates such as alcohol dehydrogenase and citrate synthase, the enzyme that catalyses the first step of the TCA cycle [34]. Given that increases in both MDH and ERD10 abundance have been observed to increase the respiration rate [29,34], an increase in MDH and ERD10 in response to cAMP implicates cAMP directly or indirectly in the modulation of the TCA cycle and, with it, energy metabolism.

In contrast, glyoxalase I, a protein that detoxifies methylglyoxal, a cytotoxic by-product of glycolysis [35], decreases in abundance in response to cAMP. In *Arabidopsis*, glyoxalase 1 (At1g08110) has been shown to decrease in abundance under abiotic stress conditions including salinity, drought, osmotic and cold [36]. Given that proteins ERD10, mitochondrial MDH and FBA3 increase and glyoxalase I decreases, we hypothesize that cAMP has a regulatory function in abiotic stress responses in general and the modulation of the TCA cycle in particular.

Of the 20 differentially expressed proteins identified in this study, five proteins, 3-oxoacyl-[acyl-carrier-protein] reductase (At1g24360), rotamase cyclophilin 1 (ROC1; At4g38740), RNA-binding protein (At2g37220), early response to dehydration 15 (ERD15; At2g41430) and mitochondrial MDH, have been associated with the response to ABA. Abscisic acid controls several aspects of development and adaptation to stress [37,38,39]. The 3-oxoacyl-[acyl-carrier-protein] reductase that catalyzes the first reduction step in fatty acid biosynthesis [40] is repressed by ABA in guard cells of *Arabidopsis* [41], but it increases in abundance in response to cAMP treatment in *Arabidopsis* cell suspension culture (Table 1). On the other hand, ROC1, a signaling protein component controlling plant responses to light, is an important link between phytochrome signaling and brassinosteroid sensitivity [42]. Rotamase cyclophilin 1 has been shown to decrease in abundance in response to abiotic stress and after ABA treatment [43], and this is consistent with the response to cAMP (Table 1).

In *Arabidopsis*, RNA-binding proteins have also been shown to play a role in ABA signaling during germination and drought tolerance [44], and ABA affects tyrosine dephosphorylation of a chloroplast-localized RNA-binding protein (At2g37220) in particular, and may modify its RNA-binding activity and thereby regulate gene expression [45]. The RNA-binding protein is related to the maize glycine-rich RNA-binding protein A, and the encoding gene has been shown to be induced by ABA and this is also a target for phosphorylation [46,47]. The protein has also been observed to increase in abundance at least two-fold in response to cold treatment [48]. However, in response to cAMP, the RNA-binding protein decreased in abundance (Table 1). It is therefore important to follow up on the phosphorylation status of this RNA-binding protein to see whether cAMP affects tyrosine dephosphorylation much like in the response to ABA.

Another protein that is involved in ABA responses is ERD15. In addition to being a negative regulator of ABA responses, ERD15 has a role in the defense against pathogens [38]. Besides the central role of ABA in controlling responses to abiotic stress stimuli, ABA also influences biotic stress responses and may interfere with signaling that is regulated by other hormones including SA [49]. ABA treatment prior to infection can increase the susceptibility of *Arabidopsis* to *Pseudomonas syringae* pv. *tomato*, while decreased ABA levels can improve SA-dependent defenses, suggesting that ABA modulates SA-dependent defense responses [50]. Expression of *ERD15* was observed to increase at least 50 times more in *Arabidopsis* inoculated with *Paenibacillus polymyxa* than in untreated plants [51]. Given that ERD15 is induced by biotic stress, rapidly but transiently induced in response to ABA [52] and increased in abundance in response to cAMP treatment, this again is consistent with the hypothesis that cAMP is part of the biotic stress response.

Glutathione *S*-transferase Φ8 (GSTF8, At2g47730), a marker for early stress and defense responses, also increases in abundance in response to cAMP (Table 1). The expression of GSTF8 can be induced by a range of biotic and abiotic stresses, hydrogen peroxide and in response to SA [53,54,55,56,57]. Overall this increase in abundance of GSTF8 upon induction by various stresses is also an indirect support for a role of cAMP in both biotic and abiotic stress responses.

The response to cAMP also leads to enrichment in the GO categories “response to cold” and “response to salt stress” (Table 1) and the proteins in these categories include the mitochondrial MDH, GSTF8, RNA-binding protein, 3-oxoacyl-(acyl-carrier-protein) reductase and ERD10. Interestingly, *ERD10* has been reported to be a general anti-stress protein that is up-regulated in response to a broad range of abiotic stresses [58] and was previously shown to increase in abundance after treatment of *Arabidopsis* leaves with 1 or 10 µM cAMP [22]. Furthermore, it is not uncommon for salinity stress-responsive proteins to also be responsive to Cd^2+^ ion stress and for both responses to confer a degree of cross-protection [59]. It is therefore not surprising to see that five out of the six proteins enriched in the category “response to salt stress” are also enriched in the category “response to cadmium ion” (Table 1).

Treatment of *Arabidopsis* roots with Cd^2+^ ions (10 µM) has been shown to alter the root proteome, e.g., an increase in the accumulation of proteins involved in the synthesis of glutathione-derived metal-binding proteins such as ATP sulfurylase, glutathione *S*-transferase, latex allergen-like proteins [60]. Interestingly, treatment of *Arabidopsis* seedlings with Cd^2+^ ions (50 µM) has been observed to increase the cellular cAMP concentration [61], and this in turn can induce the expression of enzymes involved in the phenylpropanoid pathway [62]. The phenylpropanoid pathway synthesizes precursors and metabolites protecting against abiotic stress including Cd^2+^ [63,64]. Taken together, the changing abundance of at least some proteins in response to Cd^2+^ may indeed require a direct or indirect interaction with cAMP.

What is the link between cAMP, Cd^2+^ and salinity? In *Arabidopsis*, an increase in cAMP levels significantly reduced Na^+^ influx in roots and it was shown that VIC-mediated Na^+^ currents are down-regulated by cAMP [16]. Further, treatment of *Arabidopsis* roots with 100 mM NaCl causes a decrease in cellular levels of cAMP, leading to a deactivation of protein kinase A. Thus, during salinity stress, cAMP suppresses expression of the Na^+^ efflux pump [16]. In addition, five of the differentially expressed proteins enriched in the category “response to salt stress” (Table 1) have been previously observed to undergo differential expression in response to low Cd^2+^ stress [65]. Most of the proteins enriched in “response to cadmium ion” are also enriched in the category “response to salt stress”, suggesting that cAMP has a role in both salinity and Cd^2+^ stress responses.

Twelve of the differentially expressed proteins identified (Table 1) have previously been linked to cabbage leaf curl virus infection [66]. Of these, seven show a similar differential accumulation pattern to cAMP (Table 2) consistent with a generalized role in plant defense against pathogens.

Finally, a previous study using 1 or 10 µM cAMP treatments of *Arabidopsis* leaves for 1 or 3 h implicated cAMP in light and temperature responses [22]. Despite the much higher concentration and later time points used in the earlier study, three proteins, methylesterase PCR A (At1g11580), 60S acidic ribosomal protein family (At2g27710) and RNA-binding protein (At2g37220), were common and decreased in abundance in response to cAMP, adding to the growing evidence for a key role of cAMP in the transduction and/or modulation of environmental stimuli.

## 3. Materials and Methods

### 3.1. Plant Material and Growth Conditions

*Arabidopsis thaliana* ecotype Columbia-0 (Col-0) cell suspension culture was grown in 250 mL Erlenmeyer flasks containing 100 mL of Gamborg’s B5 medium with vitamins (Sigma-Aldrich, St. Louis, MO, USA) [67] supplemented with 3% (*w*/*v*) sucrose, 0.05 µg·mL^−1^ (*v*/*v*) kinetin, 1 mg·mL^−1^ 2,4-dichlorophenoxyacetic acid and 0.05% (*w*/*v*) MES. Cells were grown in a growth chamber (Innova^®^ 43, New Brunswick Scientific Co., Edison, NJ, USA) under photosynthetic light with 12 h light/12 h dark cycles at 23 °C and orbital agitation at 120 rpm and sub-cultured every 1-days.

### 3.2. cAMP Treatment and Protein Extraction

At 10 days post-subculturing, three biological replicate flasks were treated with 100 nM of 8-bromo-cAMP and cells of each mock (equal volume of water) or cAMP treated were collected at 0, 5 and 10 min post-treatment. Media were drained off using Stericup^®^ filter unit (Millipore, Billerica, MA, USA), and the cells were immediately snap frozen in liquid nitrogen and stored at −80 °C until use. Approximately 1 g of cells was ground to a fine powder with mortar and pestle in liquid nitrogen and proteins were precipitated in trichloroacetic acid in acetone, vortexed and incubated overnight. Precipitated proteins were pelleted, washed and re-suspended in urea lysis buffer (7 M urea, 2 M thiourea, 4% (*w*/*v*) 3-[(3-Cholamidopropyl)dimethylammonio]-1-propanesulfonate). Approximately 100 µg of total soluble protein extract was reduced, alkylated, digested with trypsin and purified using Sep-Pak Vac tC18 100 mg cartridge (Waters, Milford, MA, USA), as described previously [68], prior to drying in a Speed Vac concentrator (Thermo Scientific, Bremen, Germany).

### 3.3. Peptide Labeling Using Tandem Mass Tag and Peptide Fractionation by OFFGEL Fractionator

Purified and dried tryptic peptides were labeled with tandem mass tag (TMT™) six-plex (Thermo Scientific, Bremen, Germany) according to manufacturer’s instructions. Each biological replicate was labeled separately, pooled together and then fractionated using a 3100 OFFGEL fractionator (Agilent Technologies, Santa Clara, CA, USA) using 24-well high resolution immobilized pH gradient strips, pH 3–10, as described previously [68].

### 3.4. Protein Identification by LTQ Orbitrap and Quantification of Differentially Expressed Proteins

Dried peptide fractions were re-suspended in a solution containing 5% (*v*/*v*) acetonitrile and 0.1% (*v*/*v*) formic acid and analyzed by an LTQ-Orbitrap Velos (Thermo Scientific, Bremen, Germany) coupled with a nanoelectrospray ion source (Proxeon Biosystems, Odense, Denmark) for nano-LC–MS/MS analyses. The MS scan range was 350 to 1600 *m*/*z* with the normalized collision-induced dissociation at 35.0 V. The top 10 precursor ions were selected in the MS scan with resolution R = 60,000 for fragmentation in the linear ion trap. All spectra were submitted for protein identification to MASCOT search engine (Matrix Science, London, UK) as described previously [22], except that TMT labeling was added as a fixed modification. Identified proteins were evaluated and quantitated using Scaffold Q+ software, version 4.0.4 (Proteome Software, Portland, OR, USA). Proteins were considered as positive identifications if they were identified with a minimum of two unique peptides, a MASCOT ion score ≥26, a peptide probability of 95% and a protein threshold of 99%. Abundance levels of positively identified proteins from cAMP-treated cells that were present in at least two technical replicate were compared with mock-treated cells collected at the same time point. Differential expression of a protein was considered significant if the fold change was at least ±1.5-fold change (±0.6 in log_2_ transformation) and statistical significance *p*-value of ≤0.05.

### 3.5. Computational Analysis of Functional Enrichment

The gene ontology (GO) and functional categorization analyses of the differentially expressed proteins were performed using FatiGO^+^ tool in Babelomics version 5 suite [69,70].

## 4. Conclusions

cAMP treatment causes changes in the proteome that are diagnostic for biotic and abiotic responsive proteins. Furthermore, cAMP also affects abundance levels of enzymes in the glycolytic pathway and the TCA cycle and this is likely to have direct implications for the energy-transducing pathways and ATP generation. Finally, cAMP may conceivably link biotic and abiotic stress responses in stress-dependent changes of energy metabolism.

## Figures and Tables

**Table 1:** 3′,5′-Cyclic adenosine monophosphate (cAMP)-responsive proteins after 5 and 10 min treatment.

Accession Number	Protein Name	Fold Change (Log_2_)	*p*-Value	GO Term
Proteins identified changing after 5 min of cAMP treatment				
At3g16460	Jacalin-related lectin 34	4.495	0.01358	A
At2g01140	Fructose-bisphosphate aldolase 3	3.532	0.01473	B, D, E
At1g20450	Early response to dehydration 10 (ERD10)	0.971	0.04245	A, B, E
At2g37220	RNA-binding protein	−0.607	0.00180	A, C
At1g08110	Glyoxalase I	−0.651	0.00912	B, D, E
At1g14980	Chaperonin 10	−0.733	0.02203	
At2g27710	60S acidic ribosomal protein family	−1.476	0.03411	A
Proteins identified changing after 10 min of cAMP treatment				
At1g23100	GroES-like family protein	39.566	0.02049	
At1g24360	3-Oxoacyl-[acyl-carrier-protein] reductase	9.238	0.01110	A
At1g28200	FH interacting protein 1	2.979	0.04999	
At1g14980	Chaperonin 10	1.771	0.04747	
At1g48920	Nucleolin like 1	1.434	0.03283	
At1g53240	Mitochondrial malate dehydrogenase	0.846	0.03931	A, B, C, D, E
At2g38540	Lipid transfer protein 1	0.844	0.04096	
At2g41430	Early response to dehydration 15 (ERD15)	0.756	0.03081	B, C, D
At2g47730	Glutathione *S*-transferase Φ 8	0.726	0.04412	A, B, C
At3g16450	Jacalin-related lectin 33	0.628	0.02041	A
At1g11580	Methylesterase PCR A	−0.606	0.03673	C
At4g21860	Methionine sulfoxide reductase B2	−0.697	0.04911	
At4g38740	Rotamase cyclophilin 1 (ROC1)	−0.706	0.00733	D
At5g47200	RAB GTPase homolog 1A	−0.856	0.03223	

GO, gene ontology; A, response to cold (GO:0009409); B, response to salt stress (GO:0009651); C, response to bacterium (GO:0009617); D, response to cadmium ion (GO:0046686); E, glycolytic process (GO:0006096).

**Table 2:** Comparison between proteins responsive to cAMP and proteins involved in pathogen response.

Accession Number	Protein Name	cAMP Treatment		Pathogen Response *	
		Fold Change (Log_2_)	*p*-Value	Fold Change (Log_2_)	*p*-Value
AT3G16460	Jacalin-related lectin 34	4.495	0.01358	0.274	4.90 × 10^−5^
AT2G01140	Fructose-bisphosphate aldolase 3	3.532	0.01473	0.372	1.48 × 10^−5^
AT1G20450	Early response to dehydration 10 (ERD10)	0.971	0.04245	−0.756	7.00 × 10^−6^
AT2G37220	RNA-binding protein	−0.607	0.00180	−0.539	2.35 × 10^−5^
AT2G27710	60S acidic ribosomal protein family	−1.476	0.03411	−0.692	2.25 × 10^−7^
AT1G24360	3-Oxoacyl-[acyl-carrier-protein] reductase	9.238	0.01110	−0.265	3.97 × 10^−4^
AT1G53240	Mitochondrial malate dehydrogenase	0.846	0.03931	−0.599	1.11 × 10^−5^
AT2G38540	Lipid transfer protein 1	0.844	0.04096	−2.701	5.04 × 10^−25^
AT1G11580	Methylesterase PCR A	−0.606	0.03673	−0.194	1.86 × 10^−3^
AT4G21860	Methionine sulfoxide reductase B2	−0.697	0.04911	−0.766	2.55 × 10^−8^
AT4G38740	Rotamase cyclophilin 1 (ROC1)	−0.706	0.00733	−0.827	1.40 × 10^−7^
AT5G47200	RAB GTPase homolog 1A	−0.856	0.03223	0.498	2.11 × 10^−7^

* Proteins reported in [66].

## Supplementary Materials

Supplementary materials can be found at http://www.mdpi.com/1422-0067/17/6/852/s1.
